# An NN-Based SRD Decomposition Algorithm and Its Application in Nonlinear Compensation

**DOI:** 10.3390/s140917353

**Published:** 2014-09-17

**Authors:** Honghang Yan, Fang Deng, Jian Sun, Jie Chen

**Affiliations:** 1 School of Automation, Beijing Institute of Technology, Haidian District Zhongguancun South Street No. 5, Beijing 100081, China; E-Mails: yanhonghang0803@gmail.com (H.Y.); sunjian@bit.edu.cn (J.S.); chenjie@bit.edu.cn (J.C.); 2 Key Laboratory of Intelligent Control and Decision of Complex Systems, Haidian District Zhongguancun South Street No. 5, Beijing 100081, China

**Keywords:** decomposition algorithm, data amount, Fourier neural network, nonlinear errors compensation

## Abstract

In this study, a neural network-based square root of descending (SRD) order decomposition algorithm for compensating for nonlinear data generated by sensors is presented. The study aims at exploring the optimized decomposition of data 1.00,0.00,0.00 and minimizing the computational complexity and memory space of the training process. A linear decomposition algorithm, which automatically finds the optimal decomposition of *N* subparts and reduces the training time to $\frac{1}{\sqrt{N}}$ and memory cost to $\frac{1}{N}$, has been implemented on nonlinear data obtained from an encoder. Particular focus is given to the theoretical access of estimating the numbers of hidden nodes and the precision of varying the decomposition method. Numerical experiments are designed to evaluate the effect of this algorithm. Moreover, a designed device for angular sensor calibration is presented. We conduct an experiment that samples the data of an encoder and compensates for the nonlinearity of the encoder to testify this novel algorithm.

## 1. Introduction

Compensation for nonlinear data gathered from high precision sensors is a complex task, mainly due to the data amount growth and irregular nonlinearity. It is increasingly being recognized that neural network (NN)-based compensation algorithms are widely used in linearizing the nonlinearity of many sensors and devices. Medrano-Marques *et al.* in [1] use a piecewise-linear function to approximate the nonlinear activation function of the hidden neurons and get a very similar result to those achieved with the original nonlinear activation function. Then, the NN-based model is suited for being programmed into system memory, but the precision is limited. Hafiane *et al.* in [2] use NN to learn the BTJ (buried triple PN junctions ,when a piece of semiconductor material changes type from p-type and n-type over a crosssection it forms a PN-junction.) sensor properties, and NN can produce the inverse model, which is used as a readout interface to improve sensor performance. Chiang *et al.* in [3] use a novel procedure that combines an NN architecture and RTS (Rauch-Tung-Striebel) smoother for post-mission processing, and NN helps to reach higher estimation accuracy. Lai *et al.* in [4] design NN to compensate for the pivot friction and reduce the tracking errors by 60%. José *et al.* in [5] show that the artificial neural network method has a better overall accuracy than the piecewise and polynomial linearization methods. However, as the sensors' precision becomes increasingly higher, the data obtained from the sensors increase by great speed. It is costly to employ the NN-based Levenberg-Marquardt (LM) algorithm to process the nonlinearities, since the computational complexity increases by order *O*(*n*^3^) and memory cost by order *O*(*n*^2^). Inspired by the divided-and-conquer algorithm, we design a decomposition algorithm, which breaks the data into N small portion of original data, each of which can be executed by the NN-based algorithm with less computational complexity and memory cost.

As the data amount grows, the structure of NN-based algorithms becomes complicated. Since computational complexity and memory cost grow rapidly, a decomposition algorithm is in urgent need for decreasing the computational complexity and memory cost. It has been demonstrated that finding the general optimal data distribution algorithm is a complete NP problem [6]. Many researchers proposed various data decomposition algorithms for specific issues. He *et al.* in [7] give an issue for NN-based fault diagnosis of large-scale analogue circuits. The proposed approach partitions a large-scale circuit into several small sub-circuits according to some certain rules and then tests each sub-circuit using the neural network method. Bi *et al.* in [8] give another issue of an NN-based distributed fault section estimation system. A decomposition algorithm, which is based on weighted minimum degree reordering, is proposed to partition the network into connected subnetworks, where FSE (fault section estimation) burdens and minimum frontier elements are quasi-balanced. These decomposition methods are based on the features of their special structures, and the partition technique can solve the large-scale problems. A class of issues unique to parallel architecture that has claimed to design a decomposition method and provide improvements in accelerating the training speed of NN, is presented in [9,10]. These methods are based on hardware that supports a parallel computation structure. Similar to the above methods, partition techniques in the computation process solve the large-scale problem.

The decomposition algorithm we propose partitions single NN to small-scale NNs and searches the necessary numbers of hidden nodes. We partition the data to *N* parts and search for the optimal partitioning factor *N*, for the purpose of reducing the computational complexity and memory most effectively. After being partitioned to *N* segments, the data are compensated at the same precision level by *N* smaller NN. The same order of magnitude precision of different partitioning methods is guaranteed by the finite accuracy theory. This theory is presented by Wray *et al.* in [11]. They present that the NN can be considered as constructing a polynomial approximation when the NN is implemented on a computer with finite accuracy bounds. Hidden node numbers are adjusted by the trial and error method traditionally. However, when the data amount grows, its computational complexity and memory cost grow in order *O*(*n*^3^) and *O*(*n*^2^), respectively. It is invalid to apply the trial and error method to adjust hidden node numbers. We review serial explorations of the relationship between the data and the hidden node number, searching for opportunities to lower the computational complexity and memory orders of the data. Huang *et al.* in [12] present a conservative method in the estimation of hidden node numbers for non-sigmoid NN. They rigorously prove that standard single hidden layer feedforward networks with at most *M* hidden neurons and with any bounded nonlinear activation function, which have a limit at one infinity, can learn *M* distinct samples with zero error. Tamura *et al.* in [13] give a proof showing that a three-layered feedforward network with *M* — 1 hidden units can approximate any *M* input-target relations exactly. Barron *et al.* make further progress in [14]. When the data amount is *M*, the bounds on the numbers of hidden nodes can be effectively obtained by order $O(1/\sqrt{M})$.

In order to obtain a fast training speed, we use the second order learning algorithm to train the NN. The most effective training algorithm is the LM algorithm [15-17]. Hagan *et al.* in [15] incorporate the Marquardt algorithm into the backpropagation algorithm for training feedforward NNs. It is much more efficient than either of the other techniques when the network contains no more than a few hundred weights. Ampazis *et al.* in [16] present the LM algorithm with adaptive momentum. A momentum term is added in the LM algorithm to escape from local minima. The number of hidden nodes in their test is 30. Wilamowski *et al.* in [17] optimize the NN's learning process using the LM algorithm by reducing operations in quasi-Hessian matrix calculation. These, NN-based LM algorithms are limited by the quasi-Hessian matrix. The dimension of the matrix is *n* × *n* (*n* denotes the numbers of weights, the numbers of hidden nodes). When the *n* is large, it is possible to employ the LM algorithm.

In order to utilize the power of NN, Fourier neural network (FNN) architecture is selected, because it has advantages [18-21], such as: (1) it employs orthogonal Fourier exponentials as its basis functions, determining the network structure conveniently; (2) the structure of FNN can be reconfigured according to system output information, making the learning more efficient; (3) it is possible to avoid local minimum due to the orthogonality of the basis functions; and (4) the convergence accelerator of FNN improves by a few orders of magnitude of the convergence speed.

We give several new numerical experiments to present detailed evaluations and demonstrate that the proposed method has advantages. Then, we introduce a specially designed system for compensating for nonlinear errors of angular encoders and conduct an experiment on this system to testify the effect of the SRD decomposition algorithm. The output data of the sensor with nonlinear errors are gathered, then transmitted to the PC and calibrated by the algorithm in the PC. The computational complexity and memory cost of different partitioning factor *N* of data are compared to testify to the lowering order of computational complexity and memory cost.

This paper is organized as follows. Section 2 gives the prescription of the FNN and LM training algorithm. In Section 3, we present our decomposition algorithm. In Section 4, we present several numerical experiments to present detailed evaluations of related algorithms. In Section 5, detailed information about the experimental device is given. This is followed by experimental results in Section 6. Finally, conclusions are discussed in Section 7.

## 2. FNN and LM Algorithm

In this section, we introduce the general model of FNN. We begin with Fourier series and FNN, then the LM training method is discussed.

### 2.1. Fourier Series and FNN

**Figure 1.**
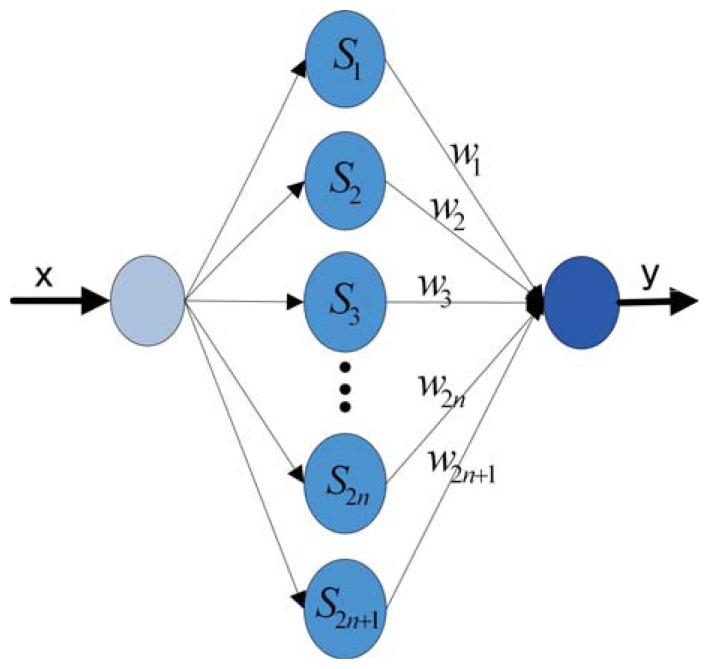
The typical structure of the Fourier neural network (FNN).

The powerful FNN can be traced back to the Fourier series. Silvescu *et al.* in [21] present that neurons of NN are capable of representing any partial Fourier series in periodic limit. The convergence accelerator of FNN improves by orders of magnitude the convergence speed. FNN is available for getting out of local minima. From the standpoint of functional analysis, the FNN can be expanded to multi-dimensions and free of periodic limit. From the definition of Fourier series of functional analysis, a function in a separable Hilbert space can be expanded in a series of orthogonal bases, namely [22]:

Assume the *H* is the Hilbert space and *S* = {*α_k_* : *k* ∈ *N*}. *S* is a standard orthogonal series. Thus, for any *x* ∈ *H*, Fourier series $\sum_{k=1}^{\infty }\langle x,\alpha_{k}\rangle \alpha_{k}\in H$, the FNN can be extended and is presented in Figure 1.

The squared error *E* is used as the error criterion and is the criterion for target precision. *E* serves as a terminate condition of the training algorithm. It is defined by the following equation:

(1) $$\begin{array}{r} E=\frac{1}{2}\sum_{1}^{M}e_{i}^{2}\\ e_{i}=y_{i}-Y_{i},i=1,2,\cdots ,M \end{array}$$

where *Y_i_* is the i-th output value of the FNN, *y_i_* is the i-th target output value and *M* is the size of the training sample.

### 2.2. The LM Method of Training NN

Adapted from the descent method and the Newton method, the LM algorithm is [7]:

(2) $$\Delta \omega ={\left(J^{T}J+\mu I\right)}^{-1}J^{T}e$$

where the *ω* is the weights of the connected neuron and I is the unit matrix. *μ* is the coefficient.

The Jacobian matrix is *J*:

(3) $$\left(\begin{array}{cccc} \frac{\partial e_{11}}{\partial \omega_{1}} & \frac{\partial e_{11}}{\partial \omega_{2}} & \cdots & \frac{\partial e_{11}}{\partial \omega_{N}}\\ \frac{\partial e_{12}}{\partial \omega_{1}} & \frac{\partial e_{12}}{\partial \omega_{2}} & \cdots & \frac{\partial e_{12}}{\partial \omega_{N}}\\ \cdots & \cdots & & \cdots \\ \frac{\partial e_{1M}}{\partial \omega_{1}} & \frac{\partial e_{1M}}{\partial \omega_{2}} & \cdots & \frac{\partial e_{1M}}{\partial \omega_{N}}\\ \cdots & \cdots & & \cdots \\ \frac{\partial e_{P1}}{\partial \omega_{1}} & \frac{\partial e_{P1}}{\partial \omega_{2}} & \cdots & \frac{\partial e_{P1}}{\partial \omega_{N}}\\ \frac{\partial e_{P2}}{\partial \omega_{1}} & \frac{\partial e_{P2}}{\partial \omega_{2}} & \cdots & \frac{\partial e_{P2}}{\partial \omega_{N}}\\ \cdots & \cdots & & \cdots \\ \frac{\partial e_{PM}}{\partial \omega_{1}} & \frac{\partial e_{PM}}{\partial \omega_{2}} & \cdots & \frac{\partial e_{PM}}{\partial \omega_{N}} \end{array}\right)$$

and the error vector is e:

(4) $$\left(\begin{array}{c} e_{11}\\ e12\\ \cdots \\ e_{1M}\\ \cdots \\ e_{P1}\\ e_{P2}\\ \cdots \\ e_{PM} \end{array}\right)$$

## 3. Decomposition Algorithm

### 3.1. Description of the Decomposition Algorithm

In this study, we propose a decomposition algorithm that uses the estimation of hidden node number and partitioning method to lower the computational complexity and memory cost order. We separate the data to small-scale data and utilize finite precision NN to process the small-scale data. Finite precision reduces the demand for the increment of hidden nodes to get infinite precision. The number of hidden nodes is determined by the trend of $n\propto \sqrt{M}$, while the complexity of the LM algorithm is closely related to n. Details are presented in the following.

#### 3.1.1. Equal-Precision Approximation of Decomposition Algorithm

Wray *et al.* in [11] demonstrate that when NNs are implemented by computers, their hidden node output functions are presented by finite polynomials in computers. Based on his ideas, we demonstrate that the different decomposition methods (partitioning factor *N* is different) obtain equal orders of magnitude precision results, since NNs are implemented on the same computer. Suppose that the hidden node nonlinearities are denoted as *p_i_*(*x*). *u* denotes the one input. *ω_i_* denotes the weight from the input to the hidden unit. *a_i_* denotes the activation of hidden node *i*. The output of the NN is represented by a sum of polynomials.

(5) $$p_{i}(x)=d_{i0r}+d_{i1r}x+d_{i2r}x^{2}+\ldots \forall i=1\ldots n$$

(6) $$\begin{array}{r} P_{u}=\sum_{i=1}^{n}c_{i}p_{i}(a_{i})\\ =\sum_{i=1}^{n}c_{i}(d_{i0}+d_{i1}\omega_{i}u+d_{i2}\omega_{i}^{2}u^{2}+\ldots )\\ =\rho_{0}+\rho_{1}u+\rho_{2}u^{2}+\ldots \end{array}$$

where $\rho_{k}=\sum_{i=1}^{n}c_{i}d_{ik}\omega_{i}^{k}$.

The polynomial function is capable of approximating any continuous function on a closed interval (Weierstrass approximating theory). Therefore, the polynomial approximation can be employed to approximate an output function. Further, the finite precision is related to the number of terms of the polynomial. The more the number of terms is, the higher the precision is and the larger the computational and memory costs it takes. Based on the above idea, nonlinear hidden nodes of FNN are denoted as *p_i_* (*x*).

The data are broken into *N* subparts. Each subpart is approximated by a small-scale FNN. We select the *j*(*j* = 1…*N*) subpart and analyze its corresponding FNN presentation. The nonlinearity of hidden node is denoted as $p_{i}^{\left(j\right)}$, and the NN is constructed of polynomial approximation:

(7) $$p_{i}^{\left(j\right)}(x)=d_{i0r}^{\left(j\right)}+d_{i1r}^{\left(j\right)}x+d_{i2r}^{\left(j\right)}x^{2}+\ldots \forall i=1\ldots n^{\left(j\right)}$$

(8) $$\begin{array}{r} P_{u}^{\left(j\right)}=\sum_{i=1}^{n^{\left(j\right)}}c_{i}p_{i}^{\left(j\right)}(a_{i})=\\ \sum_{i=1}^{n^{\left(j\right)}}c_{i}(d_{i0}+d_{i1}\omega_{i}u+d_{i2}\omega_{i}^{2}u^{2}+\ldots )\\ =\rho_{0}+\rho_{1}u+\rho_{2}u^{2}+\ldots \end{array}$$

where $\rho_{k}=\sum_{i=1}^{n(j)}c_{i}d_{ik}\omega_{i}^{k}$.

FNN presented in different decomposition methods have a united form when implemented in a computer. They are finally mapped to the polynomial form, and the differences are their coefficients. In the same computer, the computational capacity of the polynomial is the same. Therefore, hidden nodes that are presented by polynomials in the same computer have equal orders of magnitude precision.

#### 3.1.2. Estimation of Hidden Nodes

The node number in [12] is a conservative method for the upper bound. As mentioned in [14], Barron proposes a method to estimate the bound of nodes in NN. Thus, the number can be denoted as $C_{f}\times \sqrt{M}$. The coefficient *C_f_* can be calculated by the following formula:

(9) $$\begin{array}{l} f(x)=\int e^{ix}f(\omega )d\omega \\ C_{f}=\int {\left|\omega \right|}_{1}\left|f(\omega )\right|d\omega \end{array}$$

where ${\left|\omega \right|}_{1}=\sum_{j=1}^{d}{\left|\omega \right|}_{j}$ is the *l*_1_ norm of *ω* ∈ ℜ*^d^*.

In this paper, the linear function *y* = *x* is taken as the ideal output function of encoder. Calculate the Fourier representation of the form:

(10) $$\int e^{-i\omega x}f(\omega )d\omega =2\pi \times i\times (\frac{\delta (x)}{x})$$

Substitute the result to the *C_f_* formula,

(11) $$\begin{array}{r} C_{f}=\int (|\omega |f|\omega |)d\omega =\\ \int (2\pi \times i\times |\omega ∥(\frac{\delta (\omega )}{\omega })|d\omega \\ =2\pi \times \int |(\delta (\omega )|d\omega =2\times \pi \end{array}$$

In order to obtain an integer number of nodes, the π is rounded to four. Since the nodes of FNN are consisted of cos and sin symmetrically, the *C_f_* of FNN with a linear ideal output function should be eight. As is mentioned in [14], the rate of convergence is in order *O*(*M*^−1^**^/^**^2^). Thus, *C_f_* × (M^−1/2^*)* is taken as an estimate of the hidden node number.

#### 3.1.3. Partitioning Parameter of Decomposition Algorithm

The number of subpatterns, which denotes the decomposition, depends on the numerical properties. Suppose the data amount is *M* and the possible partitioning factor is *N*. The computational complexity of training FNN with the LM algorithm is: *N* × (*C_f_* × *round*(*M*/*N*))^3^, where ‘round’ means getting the integer number function. With the aim of comparing the computational complexity of different decomposition methods, the relative computational complexity demonstrates the result: *N* is determined to be six or seven according to the round of the $\sqrt{M/N}$.

(12) $$\frac{N_{1}(C_{f}{\left(\text{round}(\sqrt{\frac{M}{N_{1}}})\right)}^{3})}{N_{2}(C_{f}{\left(\text{round}(\sqrt{\frac{M}{N_{2}}})\right)}^{3})}\approx N\times ({\left(\text{round}(\sqrt{\frac{1}{N}})\right)}^{3})$$

where *N*_1_ = 1 denotes the non-partitioning method's partitioning factor and *N*_2_ denotes the decomposition method's partitioning factor; the formula compares the computational complexity of the non-partitioning method and decomposition method.

To search the optimal partitioning factor *N*, we add *N* from one to round (*M*/25). We analyze the reduction of computational complexity with the *N* increasing one by one. At the beginning, the effect is significant, for the nodes of the subpattern reduce rapidly. However, as the subpattern gets smaller and smaller, round $\left(\sqrt{M/N}\right)$ tends to be a fixed number, and the partition may lead to a swing of computational complexity. Specially, we find that the proper subpattern includes 30 ∼ 50 data. This swing period is the partitioning factor limit.

Take the 360 data to illustrate the selection process. The total number of patterns is 360. The numbers of hidden nodes are obtained by the formula $C_{f}\times \sqrt{N}$. In the case of selecting the proper number, we calculate the relative computational complexity. Table 1 (*N* denotes the separate factor; nodes denote the number of rounds of $\sqrt{N}$) shows the detailed computational complexity of different separating methods.

### 3.2. Speedup Factor Analysis

With the aim of training the FNN, the LM algorithm is applied. However, as the data amount increases, the computational complexity and memory cost increase rapidly. It is not possible for the computer to calculate and store too large of a Jacobian matrix, since the computational complexity of computing the inverse of a *n* × *n* matrix is *O*(*n*^3^) and the memory cost is *O*(*n*^2^) [23]. Therefore, the LM algorithm is limited by the data amount. However, we can partition the data into small subparts and then employ the LM algorithm to solve the problem.

Consider a target function of *M* data. In the non-partitioning method, the FNN requires $C_{f}\times \text{round}(\sqrt{M/N})$ nodes to approximate this function, where *N* = 1. Its computational complexity is:

(13) $$O({\left(C_{f}\times \text{round}(\sqrt{M/N})\right)}^{3})=O({\left(C_{f}\times \text{round}(\sqrt{M})\right)}^{3})$$

and the memory cost is:

(14) $$O({\left(C_{f}\times \text{round}(\sqrt{M/N})\right)}^{2=O({\left(C_{f}\times \text{round}(\sqrt{M})\right)}^{2})}$$

In the partitioning method, the *M* data are partitioned into *N* sub-patterns, and each subpattern has *M/N* patterns. The computational complexity of each subpart is $O({\left(C_{f}\times \text{round}(\sqrt{M/N})\right)}^{3})$, and the memory cost is $O({\left(C_{f}\times \text{round}(\sqrt{M/N})\right)}^{2})$. The total computational complexity of decomposition algorithm is:

(15) $$\begin{array}{r} N\times O({\left(C_{f}\times \text{round}(\sqrt{M/N})\right)}^{3})\dot{=}N\times (N^{-3/2})O({\left(\text{round}(\sqrt{M})\right)}^{3})\\ =N^{\left(-1/2\right)}O({\left(\text{round}(\sqrt{M})\right)}^{3}) \end{array}$$

The memory cost is:

(16) $$O({\left(C_{f}\times \text{round}(\sqrt{M/N})\right)}^{2})\dot{=}(N^{-2/2})O({\left(\text{round}(\sqrt{M})\right)}^{2})=N^{\left(-1\right)}O({\left(\text{round}(\sqrt{M})\right)}^{2})$$

Therefore, it is obvious that the decomposition algorithm can reduce the computational complexity and memory cost in order *N*^(−1/2)^ and *N*^(−1)^.

### 3.3. Algorithm Flow

**Figure 2.**
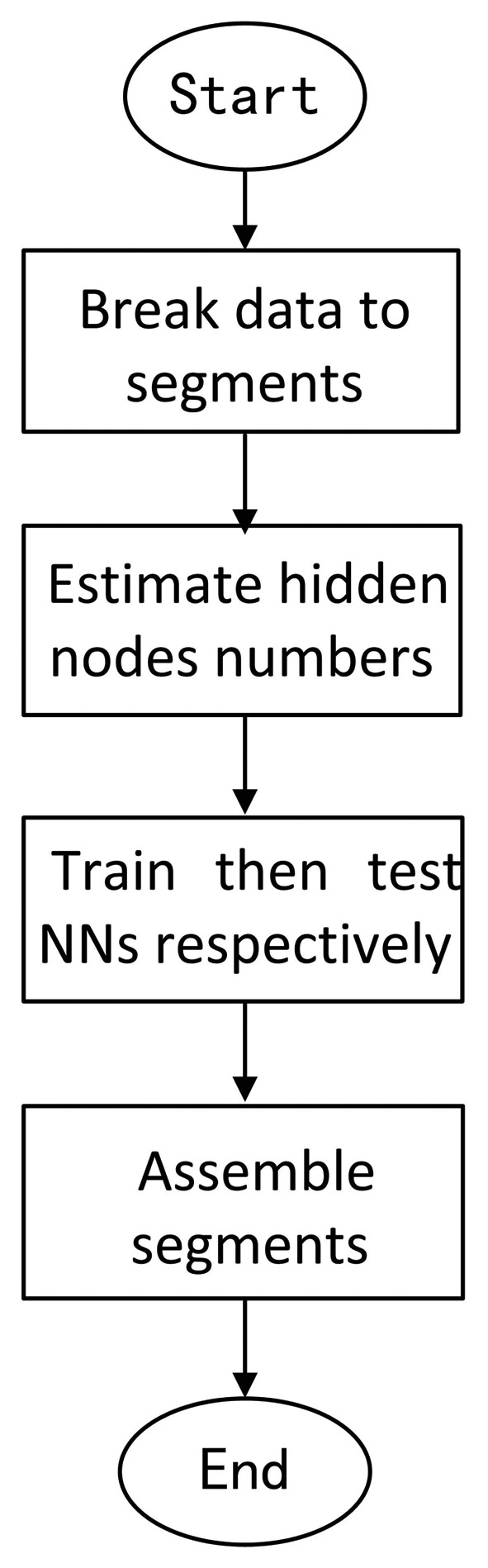
The algorithm flow.

Suppose that the data are represented by $x_{0}^{i},y_{0}^{i},i=1,2,\cdots ,M$, where the zero subscript denotes that this set of data is the training set of NN. The ideal output of an encoder is denoted by a function: *y* = *f*(*x*). Additionally, the data with subscript 1 $x_{1}^{i}$, $y_{1}^{i}$ denotes the test data. The following figure describes the flow of the algorithm (Figure 2).

Step 1: Break the data into segments
  Partitioning the data into a small-scale, on average, the number of the data in each segment is *M/N. N* is determined to be six or seven according to the round of the $\sqrt{M/N}$.
Step 2: Estimate the hidden node numbers.
  The estimation of the hidden node number is order $O(\sqrt{M/N})$. Thus, the number can be denoted as $C_{f}\times \sqrt{M/N}$. The coefficient *C_f_* can be calculated by the following formula:
  (17) $$C_{f}=\int {\left|\omega \right|}_{1}\left|f(\omega )\right|d\omega$$
  where *f*(*ω*) denotes the Fourier transformation of *f*(*x*)
Step 3: Train then test NNs, respectively.
  Employ the LM algorithm to train each segment of data, and use the trained weight to compensate for the test data.
Step 4: Assemble the segments together.
  Assemble the segments together according to the superscript.

## 4. Simulations to Evaluation and Comparison

In this section, we give several new numerical experiments to present detailed evaluations and demonstrate that the proposed method has advantages.

### 4.1. Training Algorithms Evaluation

We evaluate the backpropagation algorithm, the backpropagation with momentum algorithm and the Levenberg-Marquardt algorithm, considering the feature of iterations, average absolute error and standard deviation. The publicly available data are obtained from the test problem presented in [15]. The NN is trained to approximate the sinusoidal function $y=\frac{1}{2}+\frac{1}{4}(\sin(3\pi x))$. The training set consists of 40 input/output pairs, where the input values are in the interval [−1, 1]. Table 2 presents the result of the evaluation. As is presented in the table, the LM algorithm has the lowest average absolute error, standard deviation and a faster speed.

### 4.2. Decomposition Algorithm Evaluation (Compensation for Random Errors)

Rivera *et al.* in [5] present a linear regression model to demonstrate the evaluations. We give a linear model with random errors and employ the proposed method to compensate for the random errors. This model simulates the compensation for random errors and can demonstrate that the proposed method has advantages with publicly available data. A linear function is *y* = *x* + *e*; *x* and *y* range from [1, 2, … 360]. The input *x* is mixed with random error *e*. These errors follow Gaussian distributions (0, 0.03). Table 3 summarizes the results of this evaluation. When the partition factor *N* = 1, this method is the non-separating method, namely the traditional LM method. As is presented in Table 3, the SRD algorithm (*N* = 2, 3, 4, 5, 10) has lower training time than the original non-separating LM algorithm (*N* = 1). The standard deviation and meanabs are equal orders of magnitude precision. The SRD algorithm (*N* = 10) has the fastest training speed.

### 4.3. Decomposition Algorithm Evaluation (Compensation of Certain Trend Errors)

Hagan *et al.* in [15] present a sinusoidal wave model to demonstrate the evaluations. We give a sine wave model to demonstrate that the proposed method has advantages with publicly available data. This model simulates errors with a certain trend. The mapping function is *y* = *x* + sin(*x*/360 × 2 × π), *x* and *y* range from [1, 2, … 360]. Table 4 summarizes the results of this evaluation. When the partition factor *N* = 1, this method is the non-separating method, namely the traditional LM method. As is presented in Table 4, the SRD algorithm (*N* = 2, 3, 4, 5, 10) has lower training time than the original non-separating LM algorithm (*N* = 1). The standard deviation and meanabs are equal orders of magnitude precision. The SRD algorithm (*N* = 10) has the fastest training speed.

## 5. Experimental System

**Figure 3.**
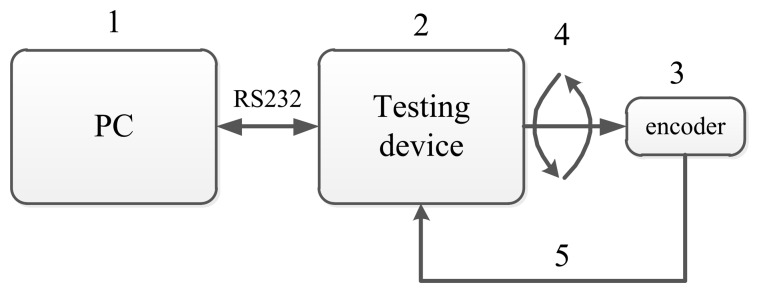
The structure of the overall system.

Deng *et al.* in [24] design a special system to compensate for and calibrate the nonlinear error of an angular sensor. It can detect less than an 18-bit different angular sensor and calibrate the nonlinear error with the NN method. The block diagram of this system for encoder calibration and compensation is given in Figure 3. This figure is a sketch map to show the components of the system. The system is mainly composed of a test device and a PC. Its mechanical transmission capacity and data transmission capacity are shown in the subsequent figures.

### 5.1. Test Device

**Figure 4.**
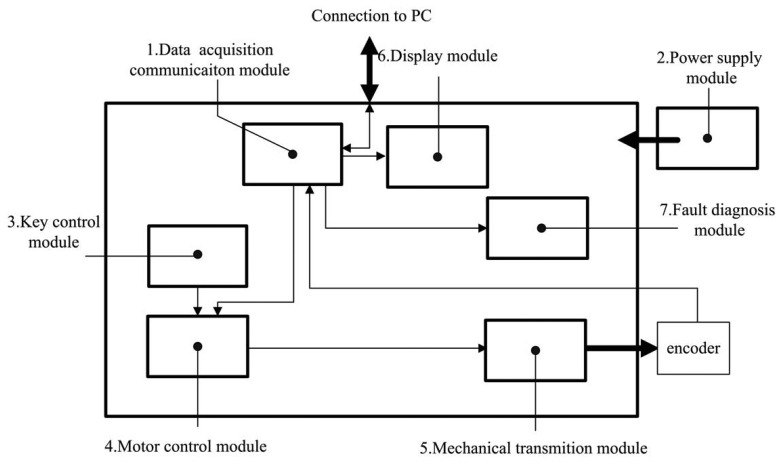
The block diagram of the test device.

The most important subsystem is the test device in Figure 4. Details of the test device are described next.

(1) Data acquisition communication module:
  The data acquisition communication module is a micro control unit (MCU). It acquires and transmits the signals of the encoder, connecting the test device and human computer interface.
(2) Power supply module:
  The power supply module provides the test device and encoder with the necessary energy to operate.
(3) Key control module:
  The key control module includes one key (red reset key) that serves as an emergency stop function. In this system, key control employs the interrupt control of MCU through the software to set the highest interrupt priority. Thus, whatever are states in which the system operates, it shall respond to the interrupt at once.
(4) Motor control module:
  The motor control module is another important part of the test device. In this module, rotation frequency, angles and the direction of the motor are controlled. The motor is controlled by its accessory motor driver. Intended to spin the motor, corresponding digital square waves are imported to the motor driver. At the rising edge of each square wave, the motor spins 0.6 degrees. Thus, the frequency of the square wave determines the frequency of rotation. The direction of the motor is determined by the shape of square waves. The above process is performed by timers of the MCU, which is capable of generating designed square waves for the target function.
(5) Mechanical transmission module:
  The mechanical transmission module requires a special design. This design requires being highly precise and reliable. Thus, under the test, the serial joint related to the motor spurs the encoder smoothly and reliably. With this purpose, flexible shaft coupling (Figure 5②④) between the motor (Figure 5①) and reduction gear (Figure 5③) are designed to guarantee steady transmission. This resolution is far higher than any reduction gear. The element of the transmission system is described next:
  (a) DC step motor: The motor in application is a step motor. Its stepping angle is 0.6 degrees.
  (b) Flexible shaft coupling: This is the joint between the motor and worm. It has the capacity of compensating for the relative offset of the axis. Thus, it obtains a cushioning and damping function.
  (c) Worm: The worm drives the worm wheel to spin.
  (d) Worm wheel: The worm wheel is driven by the worm, and its reduction relation is 108:1, which means that the worm spins once for 108 motor revolutions.
  (e) Worm-wheel shaft: The worm wheel shaft is fixed on the worm wheel and spins synchronously with the worm wheel.
  (f) Flexible shaft coupling: This flexible shaft coupling is connected to the encoder. It transmits the movement from the worm-wheel shaft to the encoder and moves the encoder under test. Due to its capacity of compensating for the axis relative offset, it protects the encoder from being damaged by emergency stop.
  According to the above description, the resolution of the described system is:
  $$\frac{0.6}{108}\approx 0.0055$$
  additionally, the precision of the encoder is:
  $$\frac{360}{65,536}=0.0055$$
  This means that when the motor steps one step, the 16-bit encoder moves one revolution.
(6) Display module:
  During the test process, the measurement data is monitored in real time, then displayed. Once a small failure occurs, the fault is maintained by the display module. By displaying the state of the system, users identify the small failure and handle it in a timely manner. It consists of LEDs and its accessory circuit. The displaying content includes real-time measurement data and a fault indicator.
(7) Fault diagnose module:
  Finally, the faults are diagnosed by the measurement data to identify the flaw of the data transmission process. By means of analyzing real-time data of the encoder, it is able to diagnose the disconnected bits of the parallel transmission line.

### 5.2. Personal Computer (PC)

**Figure 6.**
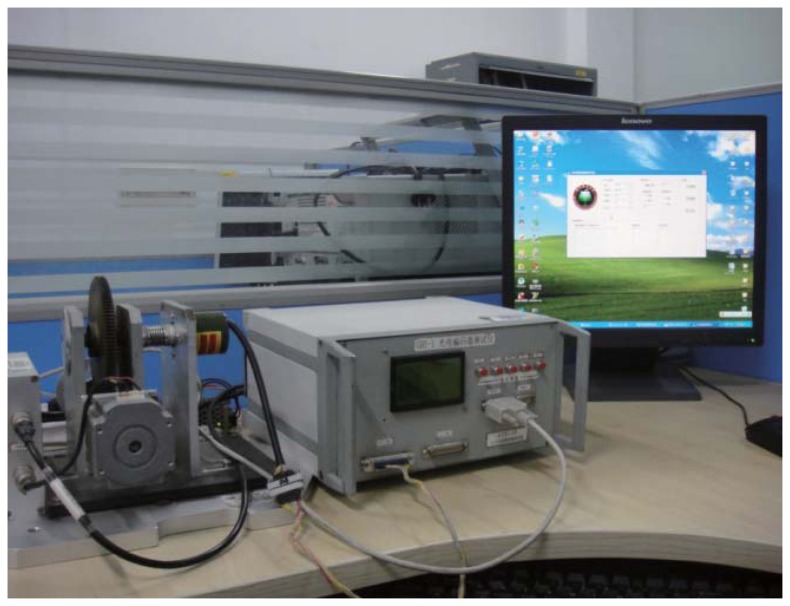
The details of the general system.

The PC is illustrated in Figure 6. It serves as the human-computer interaction, receives data transmitted from the test device and compensates for the data of the encoder by an intelligent algorithm. During the test, the encoder is spurred by the test device. With the purpose of subjecting the encoder under test to the whole range of angles, the test device spins from zero degrees to 360 degree. Thus, it obtains encoder measurement for each angle, which is processed by a data acquisition module that is acting as an interface between the test device and PC. The data of the encoder are acquired by a 16-bit parallel port with a speed up to 2 M/s. The obtained data are sent to a PC through RS232 serial communication. Then, the data are stored and shown by the human-computer interaction module. The human-computer interaction module also controls the measure-acquisition-process system.

## 6. Experiment and Analysis

The experiments are designed to show that the pre-compensation precision of the encoder improves obviously after compensation. Further, by using the same data, different separating methods are employed to train the NN, and the memory cost and computational complexity in different separating methods are compared in detail, with the aim of testifying to the theory in Section 3. The testing process of obtaining the encoder data is demonstrated primarily. Then, the compensation results of different separating methods are shown; the memory cost and computational complexity of different separating methods are compared finally.

### 6.1. Obtaining the Encoder Data Process and Preparation of Training Data

Step 1: Set the serial port, port rate, measurement time, angular intervals and detection of the motor through the human-computer interaction interface;
Step 2: Transmit the set information in packets to the controller with the communication protocol by the communication module. The controller receives the command and controls the motor according to the command. Meanwhile, the to-be-tested angular sensor is driven by the precise worm gear;
Step 3: Connect the controller and to-be-tested angular sensor to a muti-interface and transmit the position information of the sensor to the computer;
Step 4: Use the computer to deal with the detection data and analyze it, then calibrate the error.

Three hundred sixty sets of input-output patterns are obtained from the compensation system at an interval of one degree ranging from one degree to 360 degrees. Each set contains three pairs of input-output data at one degree. The first pair is the training set, and the other set is the testing set. The indicators of the data, including average absolute errors and standard deviation of the errors, are shown in the chart to represent the rate of the deviation. The output of the encoder is a nonlinear function of the input signal, while its ideal output should be a linear function of the input signal.

### 6.2. Train and Test Process and Compensation Analysis

**Figure 7.**
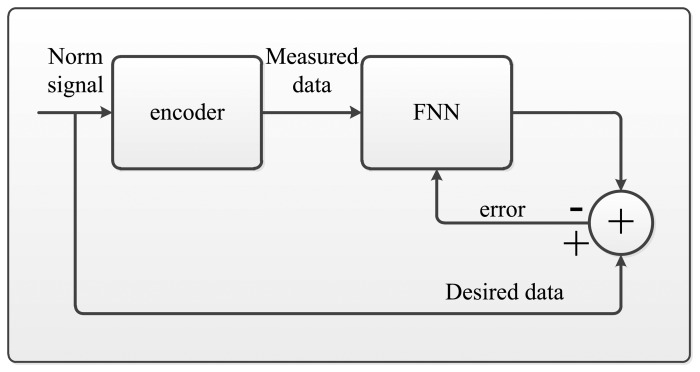
The description of the algorithm.

Figure 7 shows the mainframe of the FNN, and it is updated by the LM algorithm. In the non-separating method, the encoder outputs are taken as the input patterns, and the ideal value of the encoder outputs are taken as the desired output of the NN. Therefore, the input patterns are mapped to the FNN hidden layer with the node number calculated by the formula in Section 3. In the separating method, the input patterns and output patterns are separated into N pairs according to the separating method presented by the formula in Section 3. Each subpart of the patterns employs the non-separating method to train the corresponding NN, which has a smaller scale and less hidden nodes than the non-separating one. The weights of the FNN are adjusted by using the LM algorithm after the network is mapped with corresponding patterns. The training is terminated by the error criterion or when the epoch reaches the maximum epoch.

Once the training is processed, the adapted parameters of the FNN are loaded into the NN, until it reaches the stop condition. The FNN is evaluated by presenting both of the testing sets and the training set patterns. Then, it calculates the output after compensating. In the specification of the rotary encoder, the system precision is always presented by the standard deviation of the errors.

**Figure 8.**
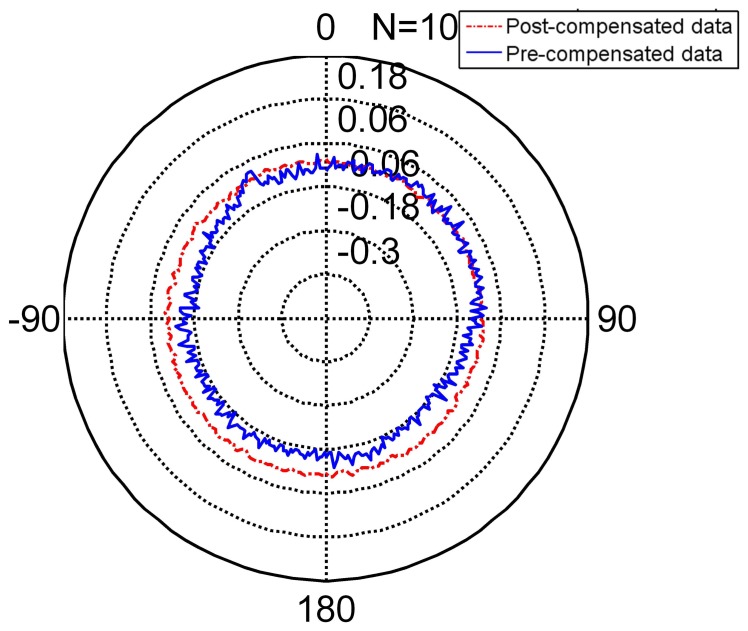
The pre-compensated and post-compensated data.

The radar Figure 8 shows the compensated data and Table 5 shows the feature of compensated data. Compared to the features in Table 6, its standard deviation reduces from (pre-compensation) to (post-compensation). It is under its resolution and achieves its nominal precision. The error range of the encoder narrows obviously. Thus, the encoder compensated for by the FNN is slightly more precise than its nominal precision.

### 6.3. Memory Cost and Computational Complexity Analysis

In this part, the memory cost and computational complexity of different separating methods are compared. The memory cost is denoted by the memory space of the Jacobian matrix. The computational complexity is demonstrated by the training time of the training process. The precision of the different separating methods is demonstrated by the average absolute errors and standard derivation errors.

**Figure 9.**
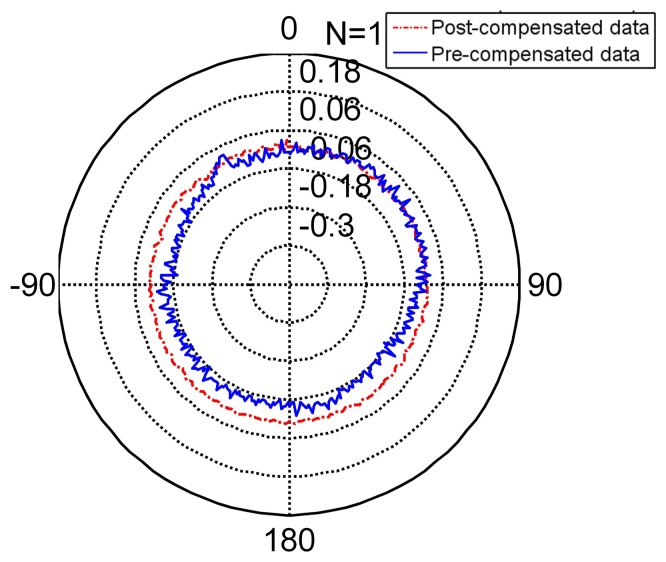
The non-separating method post-compensated data.

The radar Figure 9 presents the non-separating method of FNN in an intuitional way. The detail of its character is shown in Table 5 (TT denotes training time, MC denotes memory cost, SF denotes separating factor, Std denotes the standard deviation), Line 2. When the partition factor *N* = 1, this method is the non-separating method, namely the traditional LM method. From the derivation errors shown in the table, the derivation is under the encoder resolution and achieves its nominal precision.

**Figure 10.**
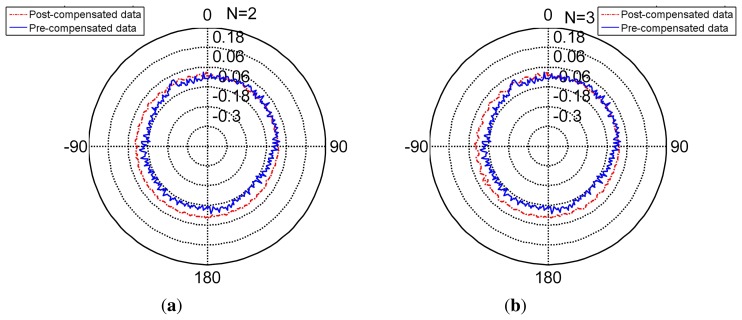
Compensation for separating factor two and three. **(a)** separating factor two; **(b)** separating factor three.

**Figure 11.**
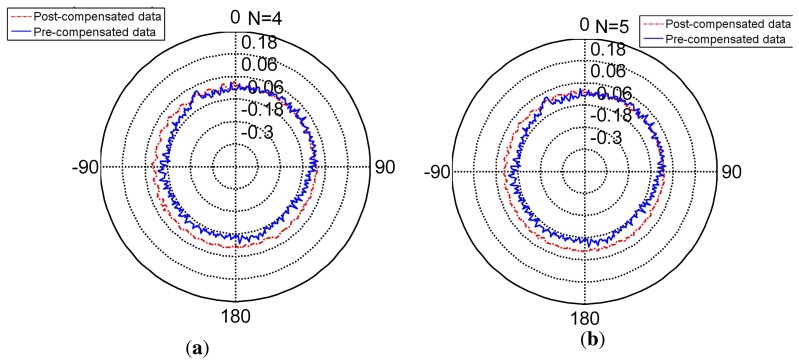
Compensation for separating factor four and five. **(a)** separating factor four; **(b)** separating factor five.

The radar Figures 8, 10 and 11 present the different separating methods, including that the separating factors are two, three, four, five and 10. The radar figure shows the results in an intuitive way, and the detail of the character is shown in the Table 5 (TT denotes training time, MC denotes memory cost, SF denotes separating factor, Std denotes the standard deviation), Lines 3, 4, 5, 6 and 7. With the aim of testifying to the theory mentioned in Section 3, comparison figures of computational cost, memory cost and precision are respectively demonstrated subsequently.

**Figure 12.**
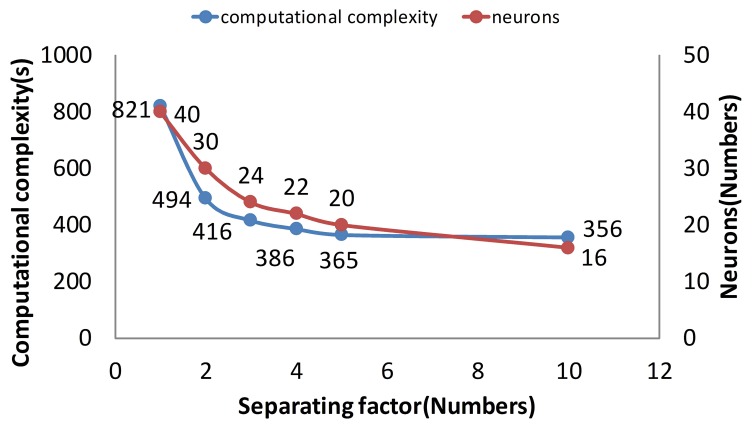
The computational complexity of different separating methods.

As is mentioned in Section 3, the core of separating is that computational complexity is reduced by the $\sqrt{N}$ trend after the separating. When the separating factor *N* is 10, the computational complexity is the lowest (Figure 12). While the separating factor *N* is one, the memory cost is the highest in the figure. Therefore, if *N* divided is employed in the training process, the training time is reduced by $\sqrt{N}$ time.

**Figure 13.**
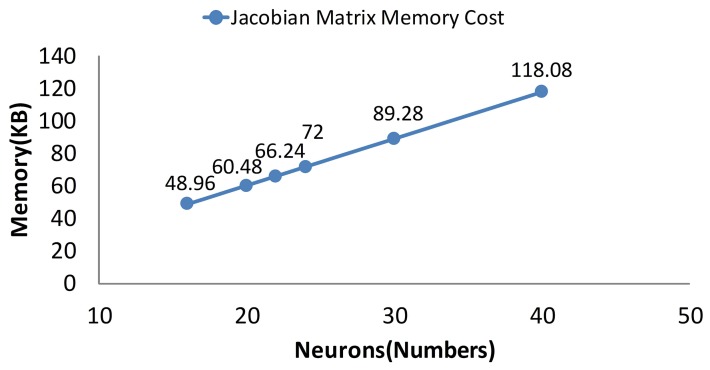
The memory cost of different separating methods.

As is mentioned in Section 3, the memory cost is reduced linearly after separating. When the separating factor *N* is 10, the memory cost is the lowest (Figure 13). While the separating factor *N* is one, the memory cost is the highest in the figure. Obviously, the memory cost is a linear trend according to the separating method.

**Figure 14.**
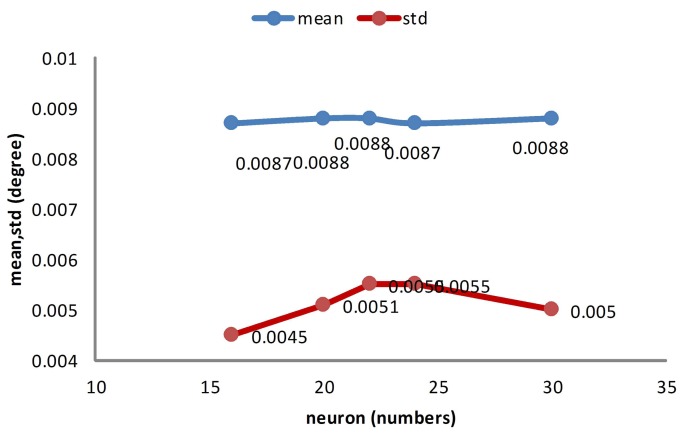
The features of post-compensated data.

Figure 14 shows the precision of different separating methods. As is shown in Figure 14, the separating factors two, three, four, five and 10 of the derivations are under resolution and achieves nominal precision. Compared to the non-separating method, these methods behave as well as the non-separating method. Further, the separating factor 10 behaves slightly better than the non-separating method.

From the above comparisons, one may notice that the separating method is slightly more effective than the traditional non-separating LM method, not only for the memory cost, but also for computational complexity.

## 7. Conclusions

In this paper, we design and evaluate an NN-based decomposition algorithm for compensating for the nonlinear data gathered by high precision sensors.

The problem of the irregular nonlinearity of errors can be solved by the NN method. The major problems of the NN method, such as the data growth amount, can be solved by the SRD algorithm. The SRD algorithm derives from the finite precision theory and hidden node estimation theory. The finite precision theory guarantees the equal orders of magnitude precision of different separating methods. The hidden node estimation theory guarantees that the SRD algorithm can reduce the computational complexity and memory cost in order *O*(*n*^−1/2^) and *O*(*n*^-1^).

The proposed method is compared against the LM method directly and the BP algorithm and the backpropagation momentum indirectly. The direct comparisons between the SRD algorithm and LM algorithm are presented in Tables 3 and 4. The standard deviation and meanabs are equal orders of magnitude precision. The SRD algorithm (*N* = 10) has the fastest training speed. The indirect comparisons are presented in Tables 2, 3 and 4. In Table 2, the standard deviation and meanabs of the LM algorithm are lower than those of the BP algorithm and the backpropagation momentum algorithm, the training speed of the LM algorithm is faster than those of the BP algorithm and backpropagation momentum algorithm. In Tables 3 and 4, the standard deviation and meanabs of SRD algorithm are equal orders of magnitude precision of the LM algorithm; the training speed of the SRD algorithm is faster than that of the LM algorithm. Therefore, the SRD algorithm behaves better than the BP and BP momentum algorithms from the indirect evaluation.

In conclusion, the proposed SRD method improves the NN training algorithm over traditional training algorithms, because it decomposes the problem of the data growth amount. Therefore, the data growth amount can be solved by the SRD algorithm.

## Figures and Tables

**Figure 5.**
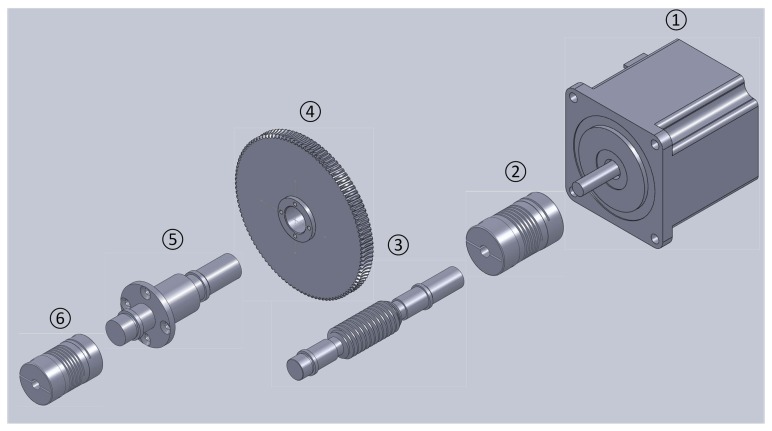
The mechanical transmission.

**Table 1.** Examples of the different separating methods.

**N (Number)**	**Nodes (Number)**	**Computational Complexity (Number)**
1	19	6859
2	14	5488
3	11	3993
4	10	4000
5	9	3645
6	9	4374
8	7	3087
9	6	2160
10	6	2592
12	5	1875
15	5	2500

**Table 2.** Training algorithms' evaluation. Std, standard deviation.

**Algorithm**	**Iterations (Steps)**	**Meanabs (Degree)**	**Std (Degree)**
Backpropagation	20,000	0.056	0.0462
Backpropagation Momentum	20,000	0.038	0.0419
Levenberg-Marquardt	32	1.2E-5	1.44E-5

**Table 3.** Decomposition algorithm evaluation of compensating for random errors.

**Partition Factor (Number)**	**Timetotal(s)**	**Std (Degree)**	**Meanabs (Degree)**
*N* = 1	656.583	1.79E-04	2.09E-04
*N* = 2	486.8203	1.89E-04	2.27E-04
*N* = 3	425.6395	1.75E-04	1.54E-04
*N* = 4	410.2213	1.85E-04	1.64E-04
*N* = 5	383.7284	1.78E-04	1.50E-04
*N* = 10	376.3153	1.88E-04	2.63E-04

**Table 4.** Decomposition algorithm evaluation of compensating for certain trend errors.

**Partition Factor (Number)**	**Timetotal (s)**	**Std (Degree)**	**Meanabs (Degree)**
*N* =1	633.3018	5.00E-04	3.88E-04
*N* =2	487.5739	3.74E-04	3.51E-04
*N* =3	399.5028	3.77E-04	3.35E-04
*N* =4	392.3897	3.58E-04	3.26E-04
*N* =5	378.7893	3.54E-04	3.19E-04
*N* = 10	373.5141	3.62E-04	3.21E-04

**Table 5.** Data features of the non-separating method for post-compensated data (TT denotes training time, MC denotes memory cost, KB denotes one thousand bits, SF denotes separating factor, Std denotes the standard deviation).

**SF (Number)**	**TT (s)**	**MC (KB)**	**Nodes (Numbers)**	**Std (Degree)**	**Mean (Degree)**
1	821	118	40	0.0046	0.0088
2	494	89	30	0.005	0.0088
3	416	72	24	0.0055	0.0087
4	386	66	22	0.0055	0.0088
5	365	60	20	0.0051	0.0088
10	356	49	16	0.0045	0.0087

**Table 6.** The data features of pre-compensation.

**Items**	**Meanabs (Degree)**	**Std (Degree)**
Data	0.245	0.241
